# Associations of T helper 1, 2, 17 and regulatory T lymphocytes with mortality in severe sepsis

**DOI:** 10.1007/s00011-013-0630-3

**Published:** 2013-05-14

**Authors:** Huang-Pin Wu, Kong Chung, Chun-Yao Lin, Bor-Yiing Jiang, Duen-Yau Chuang, Yu-Chih Liu

**Affiliations:** 1 Division of Pulmonary, Critical Care and Sleep Medicine, Chang Gung Memorial Hospital, Keelung 222, Maijin Rd, Anle Chiu, Keelung, 204 Taiwan ROC; 2 Department of Emergence, Chang Gung Memorial Hospital, Keelung, 204 Taiwan; 3 Department of Chemistry, National Chung-Hsing University, Taichung, 402 Taiwan; 4 Chang Gung University College of Medicine, 333 Taoyuan, Taiwan

**Keywords:** T helper 1 lymphocytes, Regulatory T lymphocytes, T helper 17 lymphocytes, Severe sepsis, Mortality

## Abstract

**Objective and design:**

T helper 17 (Th17) and regulatory T (T_reg_) lymphocytes might play important roles in patients with severe sepsis. The association of Th17 or T_reg_ lymphocytes with survival is also unclear.

**Methods:**

Eighty-seven patients with severe sepsis were enrolled from our intensive care units between August 2008 and July 2010. Leukocyte antigens and clinical data were determined on day 1 in all patients and on day 7 in first-year patients.

**Results:**

The percentages in peripheral blood mononuclear cells (PBMCs) and circulatory counts of CD4^+^ and CD8^+^ lymphocytes in survivors were higher than those in non-survivors. Th1/CD4^+^ ratios and circulatory Th1 lymphocyte counts in survivors were higher than in non-survivors. Absolute counts of Th17 and T_reg_ lymphocytes in survivors were higher than in non-survivors. The percentages of CD4^+^ and CD8^+^ in survivors’ PBMCs were increased after 6 days. Th17/CD4^+^ ratios and circulatory Th17 lymphocyte counts in survivors were increased after 6 days.

**Conclusions:**

Higher Th1 differentiation and total CD4^+^ T lymphocyte counts were associated with higher survival. The association of circulatory Th17 and T_reg_ lymphocytes with mortality in severe sepsis may be due to the change in total CD4^+^ T lymphocytes. In survivors, Th17 differentiation and counts were restored.

## Introduction

The majority of clinical and basic studies on sepsis have focused on the balance between the function of T helper 1 (Th1) and Th2 cells. The presence of interleukin (IL)-12 with signaling through signal transduction and activator of transcription (STAT)-4 skews towards Th1, whereas the presence of IL-4 with signaling through STAT-6 skews towards Th2. Th1 cells generally secrete pro-inflammatory cytokines such as interferon (IFN)-γ, tumor necrosis factor (TNF)-α and IL-1β, and Th2 cells generally have an important impact on allergic/atopic disorders and the humoral immune response. In patients with sepsis, the median Th1/Th2 ratio was lower [1]. This suggests that activated T helper cells evolve from the Th1 phenotype to the Th2 phenotype in patients with severe sepsis or that patients with low Th1/Th2 ratios develop sepsis more easily.

However, naïve CD4^+^ T helper cells can be induced to differentiate towards Th1, Th2, Th17 and regulatory T (T_reg_) phenotypes according to the local cytokine milieu [2]. The presence of IL-6 and transforming growth factor (TGF)-β skews towards Th17 [3], and that of TGF-β skews towards T_reg_ cells [4]. Th17 cells can produce IL-17, which acts in vitro and in vivo as a potent inflammatory cytokine [5]. T_reg_ cells have several subsets, including naturally occurring T_reg_ cells, induced T_reg_ cells, Tr1 cells and Th3 cells with CD4^+^CD25^+^ expression [6, 7].

Tr1 cells produce high levels of IL-10 and Th3 cells secrete large amounts of TGF-β. In addition, T_reg_ cells have the ability to suppress the proliferation of T cells [8]. In a mouse model of sepsis, adoptive transfer of in-vitro-stimulated T_reg_ cells significantly improved bacterial clearance, which resulted in improved survival [9]. This was associated with increased TNF-α production in the peritoneum. In another mouse model, the administration of anti-CD25 antibody 3 days before sepsis induced by cecal ligation and puncture reduced the early lethality [10]. These conflicting results indicate that the precise mechanism involved in sepsis remains to be established. In contrast to the traditional concept of Th1/Th2 balance, a new model of pro-inflammatory cells (Th1 and Th17) and anti-inflammatory cells (Th2 and T_reg_) is emerging.

The role of Th17 and T_reg_ lymphocytes in the pathogenesis of severe sepsis is unknown. The association of Th17 or T_reg_ lymphocytes with survival or septic shock is also unclear. Thus, we designed this study to determine the expressions of Th17 and T_reg_ cells in patients with severe sepsis.

## Materials and methods

### Participants

From August 2008 to July 2010, 87 patients who were admitted to our intensive care unit (ICU) due to severe sepsis were enrolled into this study. Patients with high-dose steroids, immunosuppressive medication or post-chemotherapy were excluded. To validate the experimental findings, 30 persons who visited our health evaluation center for examinations were enrolled as healthy controls. Severe sepsis was defined according to the consensus criteria [11, 12]. Systemic inflammatory response syndrome (SIRS) was defined as two or more of the following criteria: (1) body temperature >38 °C or <36 °C; (2) respiratory rate >20 breaths/min; (3) heart rate >90 beats/min; and (4) white blood count (WBC) >12,000/μl or <4,000/μl or >10 % bands. Sepsis was defined as SIRS with infection. Severe sepsis was defined as sepsis with one or more sepsis-induced organ dysfunctions, such as shock, respiratory failure, acute renal failure, jaundice and thrombocytopenia. Septic shock was defined as sepsis-induced hypotension (mean artery pressure <70 mmHg) unresponsive to fluid resuscitation. Respiratory failure was defined as ventilation dysfunction with the need of invasive ventilator support. Acute renal failure was defined as a rapid increase in creatinine level (>0.5 mg/dl). Jaundice was defined as hyperbilirubinemia (total bilirubin >2 mg/dl). Thrombocytopenia was defined as a platelet count below 150,000/μl. Disease severity was assessed with the acute physiology and chronic health evaluation (APACHE) II score [13]. Standard treatment according to guidelines (Surviving Sepsis Campaign) was provided to all patients [12, 14]. The informed consent was provided from a close family member. This investigation (96-1465B, 97-2179B, 98-3511B) was approved by the Institutional Review Board at Chang Gung Memorial Hospital. Patients who survived for more than 28 days after admission to our ICU were defined as survivors. All co-morbidities and past histories of the subjects were recorded.

### Peripheral blood mononuclear cell (PBMC) preparation

Whole blood (10 ml) was obtained within 48 h of admission to the ICU at 08:00 a.m. from each subject and immediately mixed with heparin. The day of first blood sampling was defined as day 1. Patients enrolled from July 2008 to June 2009 were sampled again after 6 days. The PBMCs were isolated via differential centrifugation over Ficoll-Plaque (Amersham Biosciences, Uppsala, Sweden) from whole blood within 2 h of collection.

### Flow cytometric analysis of PBMCs

**Fig. 1:**
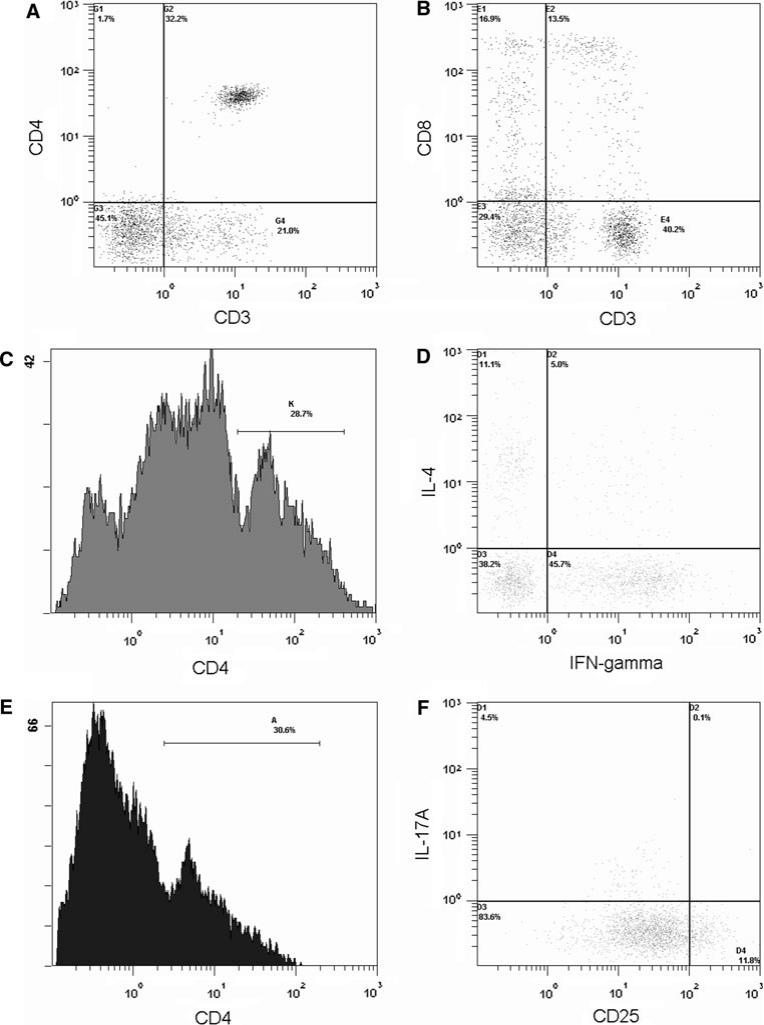
Flow cytometry of cells from a sepsis patient. CD4 lymphocytes were identified with positive CD3 and CD4 (**a**). CD8 lymphocytes were identified with positive CD3 and CD8 (**b**). Cells in **d** are gated in area K in **c**. Th1 cells were identified with positive CD4 and IFN-γ, and negative IL-4 (**d**). Th2 cells were identified with positive CD4 and IL-4, and negative IFN- γ. Cells in **f** are gated in area A in **e**. Th17 cells were identified with positive CD4 and IL-17, and negative CD25 (**f**). Regulatory T cells were identified with positive CD4 and high CD25, and negative IL-17

2.5 × 10^5^ PBMCs were suspended in 50 μl of phosphate-buffered saline (PBS) and incubated in the dark for 15 min at room temperature with 20 μl of Tritest reagent (CD4FITC, CD8PE, CD3PerCP) (Becton–Dickinson, CA, USA). The cells were then resuspended in 500 μl of PBS. The CD4 (CD3^+^CD4^+^) and CD8 (CD3^+^CD8^+^) lymphocytes were detected by a three-color flow cytofluorimeter (Beckman Coulter, CA, USA) (Fig. 1a, b).

2.5 × 10^5^ PBMCs were stimulated in vitro by 5 μg/ml phytohemagglutinin (PHA) in 1 ml sterile RPMI1640 (Gibco, Grand Island, USA) tissue culture medium containing 5 % heat-inactivated bovine serum and 1 mM sodium pyruvate (Gibco). The cells were cultured for 4 h at 37 °C and 5 % CO_2_. The cells were then suspended in 50 μl of PBS and incubated in the dark for 15 min at room temperature with 20 μl of CD4PerCP antibody. The cells were added to 500 μl of permeabilizing solution (Becton–Dickinson, CA, USA) and 10 μg/ml of brefeldin A (Sigma, Missouri, USA). The cells were incubated for 10 min in the dark at room temperature after vortexing. The cells were washed with PBS containing 0.5 % bovine serum albumin and 0.1 % NaN_3_, and then incubated with antibodies (anti-IFN-γFITC and anti-IL-4PE) (Becton–Dickinson). Finally, the cells were resuspended in 500 μl of PBS. Type 1 Th1-like (CD4^+^IFN-γ^+^) and Th2-like (CD4^+^IL-4^+^) lymphocytes were detected by a three-color flow cytofluorimeter (Beckman Coulter) (Fig. 1c, d).

2.5 × 10^5^ PBMCs were stimulated in vitro by 5 μg/ml PHA in 1 ml sterile RPMI1640 (Gibco) tissue culture medium containing 5 % heat-inactivated bovine serum and 1 mM sodium pyruvate (Gibco). The cells were cultured for 4 h at 37 °C and 5 % CO_2_. The cells were then suspended in 50 μl of PBS and incubated in the dark for 15 min at room temperature with 20 μl of CD4PerCP and CD25FITC antibodies (Becton–Dickinson). The cells were added to 500 μl of permeabilizing solution and 10 μg/ml of brefeldin A. The cells were incubated for 10 min in the dark at room temperature after vortexing. The cells were washed with PBS containing 0.5 % bovine serum albumin and 0.1 % NaN_3_, and then incubated with antibodies (anti-IL-17APE) (eBioscience, CA, USA). Finally, the cells were resuspended in 500 μl of PBS. According the references [15, 16], CD4^+^CD25^+high^ T cells were considered as T_reg_ cells. T_reg_ (CD4^+^CD25^+high^) and Th17 (CD4^+^IL-17A^+^) lymphocytes were detected by a three-color flow cytofluorimeter (Beckman Coulter) (Fig. 1e, f).

### Calculation of absolute cell counts

Absolute cell counts were the sum of total monocytes and lymphocytes times each cell percentage of PBMCs. Total monocytes and lymphocytes were obtained from WBC and WBC differential counts in the hospital hematologic study.

### Statistical analysis

Statistical analysis was performed with SPSS software v11.0.1 for Windows (SPSS Inc., IL, USA). The Kolmogorov–Smirnov test was used to test normal distribution. Differences in continuous variables between two groups were analyzed by the Mann–Whitney test. Differences in categorical variables between two groups were compared by the chi-squared test or Fisher’s exact test. Differences in continuous variables in the same subjects were analyzed using the Wilcoxon signed-rank test. A *p* value less than 0.05 was considered statistically significant.

## Results

Table 1 shows the clinical characteristics among survivors, non-survivors and healthy controls. The adverse event percentage of jaundice in non-survivors was significantly higher than that in survivors. There were no differences in percentages of new arrhythmia, gastrointestinal bleeding, acute renal failure, shock, thrombocytopenia and bacteremia. The APACHE II score in non-survivors was higher than that in survivors. Age, male/female ratio, histories, infection source and susceptibility of initial antibiotics for pathogens in survivors were similar to those in non-survivors. Age and WBC in healthy controls were significantly lower than those in survivors and non-survivors. Lymphocyte percentage and lymphocyte count in healthy controls were significantly higher than those in survivors and non-survivors.

**Table 1:** Clinical characteristics of survivors, non-survivors and controls

	Survivors (*n* = 60)	Non-survivors (*n* = 27)	Controls (*n* = 30)
Age (years)	71.6 ± 1.8	74.8 ± 2.0	60.7 ± 1.9^*†^
Males	37 (62 %)	15 (56 %)	22 (73)
APACHE II score	24.8 ± 0.9	29.5 ± 1.4^*^	
WBC (/μl)	16,351.7 ± 863.7	16,063.0 ± 1667.4	5,783.3 ± 281.1^*†^
Lymphocytes (%)	8.5 ± 1.2	10.4 ± 2.5	38.3 ± 2.2^*†^
Lymphocytes (/μl)	1,233.2 ± 154.1	1,553.3 ± 435.4	2,140.3 ± 121.6^*†^
History			
COPD	12 (20 %)	5 (19 %)	
Heart failure	5 (8 %)	0 (0 %)	
Pneumoconiosis	3 (5 %)	3 (11 %)	
Bronchiectasis	2 (3 %)	0 (0 %)	
Hypertension	25 (42 %)	9 (33 %)	
Diabetes mellitus	22 (37 %)	9 (33 %)	
Previous CVA	22 (37 %)	7 (26 %)	
End stage renal disease	6 (10 %)	4 (15 %)	
Liver cirrhosis	2 (3 %)	4 (15 %)	
Active malignancy	5 (8 %)	6 (22 %)	
Infection source			
Pneumonia	51 (85 %)	22 (81 %)	
Urinary tract infection	7 (12 %)	0 (0 %)	
Others	2 (3 %)	5 (19 %)	
Initial antibiotics for pathogens			
Sensitive	32 (53 %)	16 (59 %)	
Resistant	16 (27 %)	11 (41 %)	
No pathogen isolated	12 (20 %)	0 (0 %)	
Adverse event			
New arrhythmia	4 (7 %)	3 (11 %)	
Gastrointestinal bleeding	12 (20 %)	4 (15 %)	
Acute renal failure	25 (42 %)	16 (59 %)	
Shock	34 (57 %)	21 (78 %)	
Thrombocytopenia	16 (27 %)	12 (44 %)	
Jaundice	2 (3 %)	5 (19 %)^*^	
Bacteremia	12 (20 %)	8 (30 %)	

Data are presented as number (%) or mean ± standard error of the mean

*APACHE* Acute Physiology and Chronic Health Evaluation, *WBC* white blood cell count, *COPD* chronic obstructive pulmonary disease, *CVA* cerebral vascular accident

^*^ *p* < 0.05 compared with survivors by the Mann–Whitney test

^†^ *p* < 0.05 compared with non-survivors by the Mann–Whitney test

### Cell analysis among survivors, non-survivors and controls

**Fig. 2:**
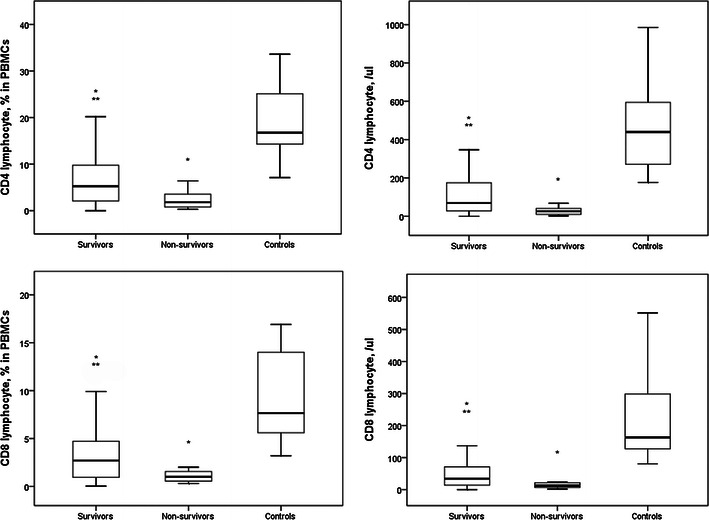
The percentages of CD4^+^ and CD8^+^ lymphocytes in PBMCs of survivors were significantly higher than that of non-survivors. The percentages of CD4^+^ and CD8^+^ lymphocytes in PBMCs of survivors and non-survivors were significantly lower than that of controls. The circulatory counts of CD4^+^ and CD8^+^ lymphocytes in survivors were also higher than that in non-survivors. The circulatory counts of CD4^+^ and CD8^+^ lymphocytes in survivors and non-survivors were lower than that in controls. *p < 0.05 compared with controls, **p < 0.05 compared with non-survivors

The percentages of CD4^+^ and CD8^+^ lymphocytes in PBMCs of survivors were significantly higher than those of non-survivors (Fig. 2). The percentages of CD4^+^ and CD8^+^ lymphocytes in PBMCs of survivors and non-survivors were significantly lower than those of controls. The circulatory counts of CD4^+^ and CD8^+^ lymphocytes in survivors were also higher than those in non-survivors. The circulatory counts of CD4^+^ and CD8^+^ lymphocytes in survivors and non-survivors were lower than those in controls.

**Fig. 3:**
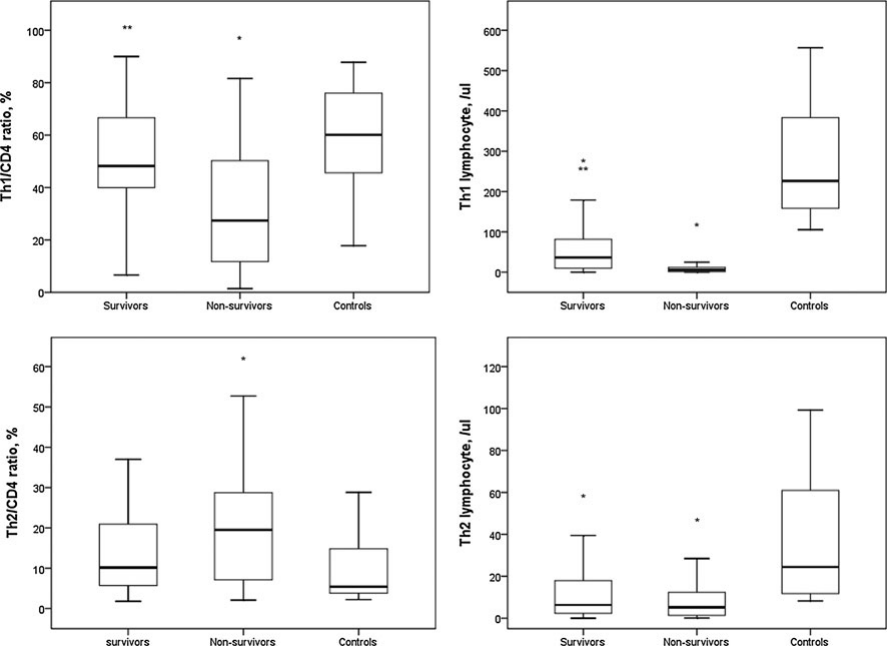
Th1/CD4^+^ ratios and circulatory Th1 lymphocytes counts were significantly higher in survivors than in non-survivors. Th1/CD4^+^ ratios and circulatory Th1 lymphocytes counts were lower in non-survivors than in controls. Circulatory Th1 lymphocytes counts in survivors were lower than in controls and there was no difference in Th1/CD4^+^ ratio between survivors and controls. Th2/CD4^+^ ratio in non-survivors was higher than in controls. Although median Th2/CD4^+^ ratio in non-survivors was higher than in survivors, the difference was not significant. The difference in Th2/CD4^+^ ratio was similar between survivors and controls. Circulatory Th2 lymphocyte counts in survivors and non-survivors were lower than in controls. Circulatory Th2 lymphocyte counts were similar between survivors and non-survivors. *p < 0.05 compared with controls, **p < 0.05 compared with non-survivors

Th1/CD4^+^ ratios and circulatory Th1 lymphocytes counts were significantly higher in survivors than in non-survivors (Fig. 3). Th1/CD4^+^ ratios and circulatory Th1 lymphocytes counts were lower in non-survivors than in controls. Circulatory Th1 lymphocytes counts in survivors were lower than those in controls and there was no difference in Th1/CD4^+^ ratios between survivors and controls. Th2/CD4^+^ ratios in non-survivors were higher than those in controls. Although the median Th2/CD4^+^ ratio in non-survivors was higher than that in survivors, the difference was not significant. The difference in Th2/CD4^+^ ratio was similar between survivors and controls. Circulatory Th2 lymphocyte counts in survivors and non-survivors were lower than those in controls. Circulatory Th2 lymphocyte counts were similar between survivors and non-survivors.

**Fig. 4:**
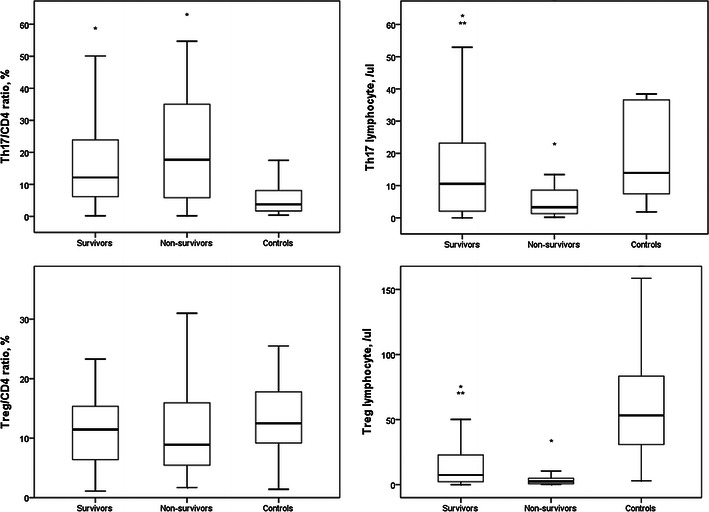
Compared with non-survivors, survivors had significantly higher circulatory counts of Th17 and T_reg_ lymphocytes. Th17/CD4^+^ and T_reg_/CD4^+^ ratios in survivors were similar to those in non-survivors. Th17/CD4^+^ ratio and circulatory Th17 lymphocyte counts in survivors and non-survivors were higher and lower, respectively, than in controls. There was no difference in T_reg_/CD4^+^ ratio among survivors, non-survivors and controls. T_reg_ lymphocyte counts in survivors and non-survivors were lower than in controls. *p < 0.05 compared with controls, **p < 0.05 compared with non-survivors

Compared with non-survivors, survivors had significantly higher circulatory counts of Th17 and T_reg_ lymphocytes (Fig. 4). Th17/CD4^+^ and T_reg_/CD4^+^ ratios in survivors were similar to those in non-survivors. Th17/CD4^+^ ratios and circulatory Th17 lymphocyte counts in survivors and non-survivors were higher and lower, respectively, than those in controls. There was no difference in T_reg_/CD4^+^ ratios among survivors, non-survivors and controls. T_reg_ lymphocyte counts in survivors and non-survivors were lower than those in controls.

### Cell analysis in survivors and non-survivors between day 1 and day 7

**Fig. 5:**
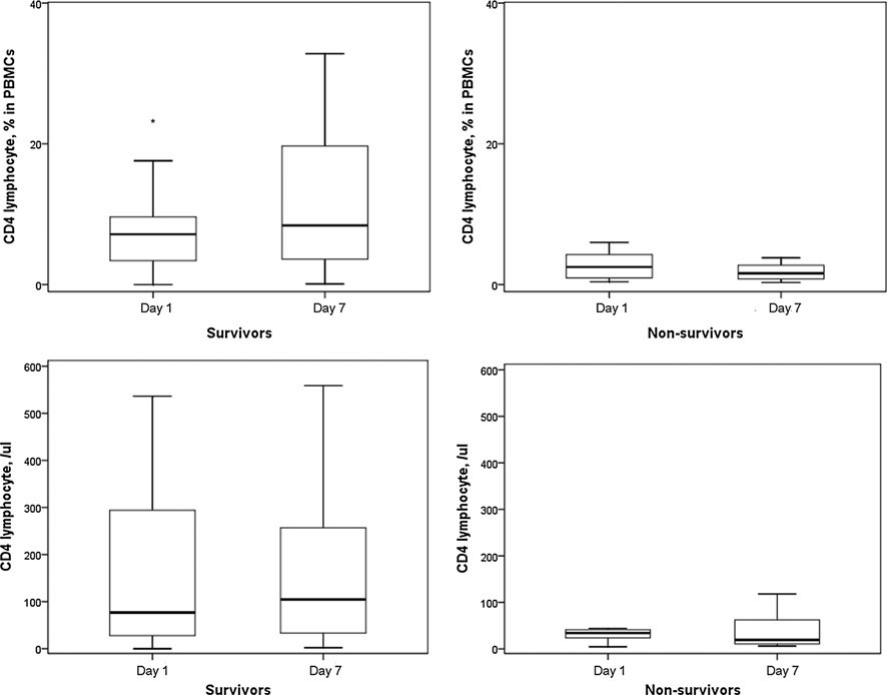
The percentages of CD4^+^ lymphocytes in PBMCs of survivors were significantly increased after 6 days. However, circulatory CD4^+^ lymphocyte counts in survivors did not increase after 6 days. The CD4^+^ lymphocyte percentages and counts in non-survivors did not change after 6 days. *p < 0.05 compared with day 7

**Fig. 6:**
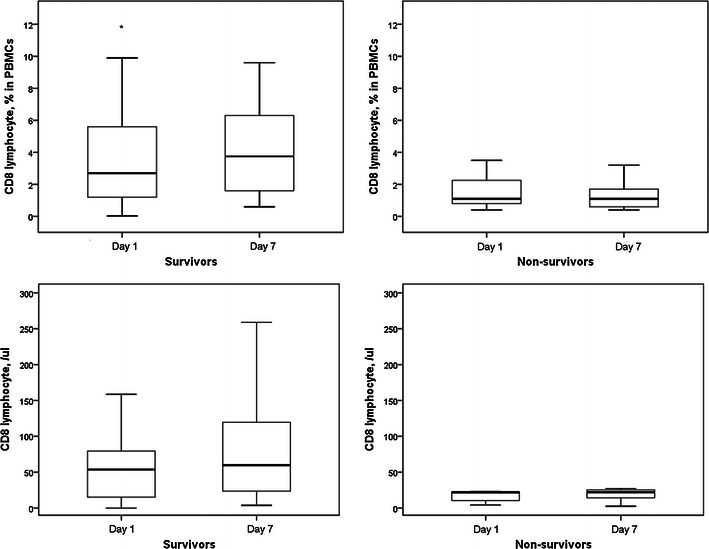
The percentages of CD8^+^ lymphocytes in PBMCs of survivors were significantly increased after 6 days. Circulatory CD8^+^ lymphocyte counts in survivors did not increase after 6 days. The CD8^+^ lymphocyte percentages and counts in non-survivors did not change after 6 days. *p < 0.05 compared with day 7

From July 2008 to August 2009, 35 patients were enrolled. Twenty-three patients survived, six patients died with 7 days, and six patients died within 8–28 days. The percentages of CD4^+^ lymphocytes in PBMCs of survivors were significantly increased after 6 days (Fig. 5). However, circulatory CD4^+^ lymphocyte counts in survivors did not increase after 6 days. The CD4^+^ lymphocyte percentages and counts in non-survivors did not change after 6 days. The percentages of CD8^+^ lymphocytes in PBMCs of survivors were significantly increased after 6 days (Fig. 6). Circulatory CD8^+^ lymphocyte counts in survivors did not increase after 6 days. The CD8^+^ lymphocyte percentages and counts in non-survivors did not change after 6 days.

**Fig. 7:**
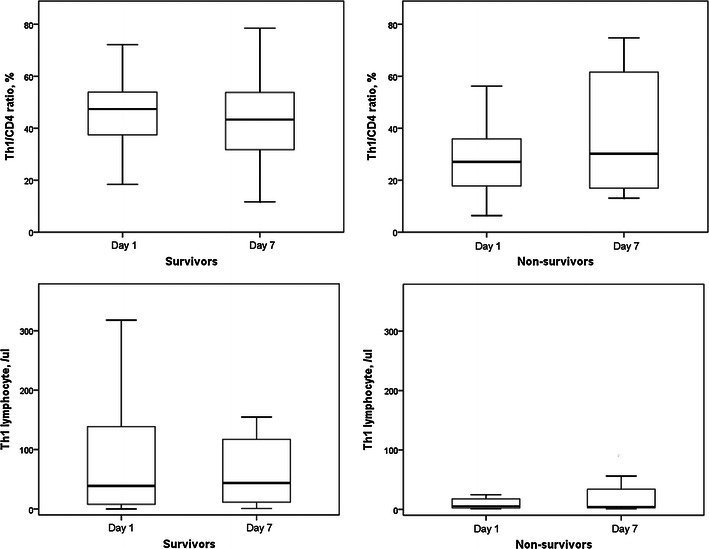
Th1/CD4^+^ ratios and circulatory Th1 lymphocyte counts in survivors and non-survivors did not change after 6 days

**Fig. 8:**
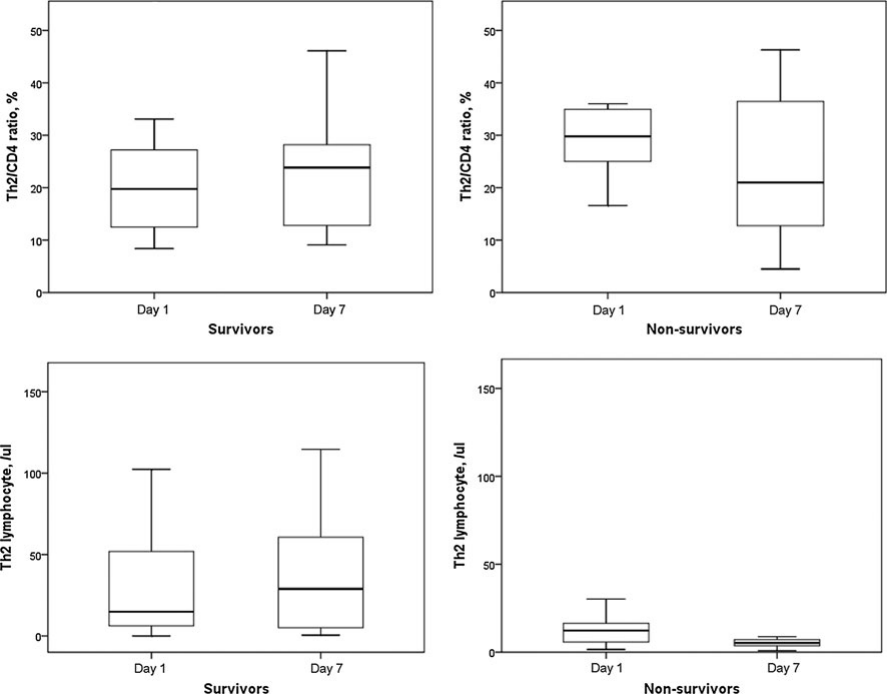
Th2/CD4^+^ ratios and circulatory Th2 lymphocyte counts in survivors and non-survivors did not change after 6 days

**Fig. 9:**
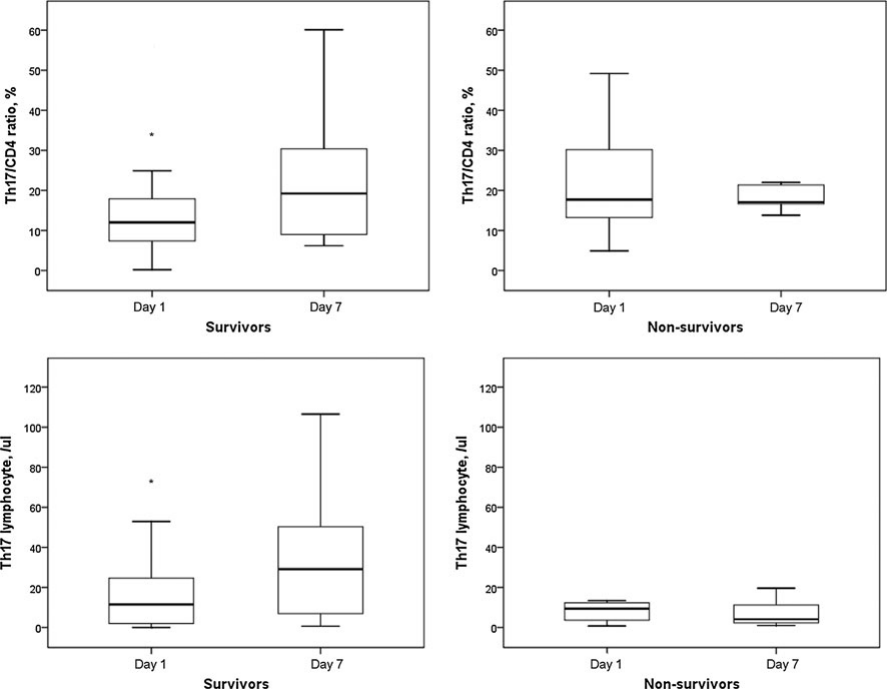
Th17/CD4^+^ ratios and circulatory Th17 lymphocyte counts in survivors were significantly increased after 6 days. Th17/CD4^+^ ratios and circulatory Th17 lymphocyte counts were similar between day 1 and day 7 in non-survivors. *p < 0.05 compared with day 7

**Fig. 10:**
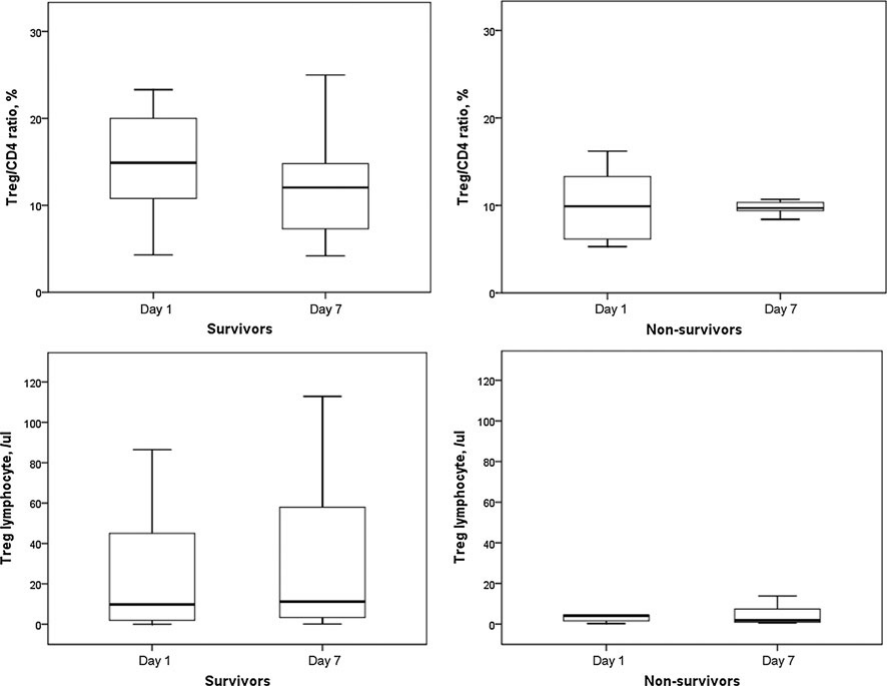
T_reg_/CD4^+^ ratios and T_reg_ lymphocyte counts in survivors and non-survivors did not change after 6 days

Th1/CD4^+^ ratios and circulatory Th1 lymphocyte counts in survivors and non-survivors did not change after 6 days (Fig. 7). Th2/CD4^+^ ratios and circulatory Th2 lymphocyte counts in survivors and non-survivors also did not change after 6 days (Fig. 8). However, Th17/CD4^+^ ratios and circulatory Th17 lymphocyte counts in survivors were significantly increased after 6 days (Fig. 9). Th17/CD4^+^ ratios and circulatory Th17 lymphocyte counts between day 1 and day 7 were similar in non-survivors. T_reg_/CD4^+^ ratios and T_reg_ lymphocyte counts in survivors and non-survivors did not change after 6 days (Fig. 10).

## Discussion

We found that the circulatory T_reg_ lymphocyte counts on day 1 in survivors with severe sepsis were higher than those in non-survivors. This result is similar to the study of Hein et al. [17], who found that survivors with septic shock had consistently higher T_reg_ lymphocyte counts than non-survivors. These findings indicate a specific role of T_reg_ lymphocytes in patients with severe sepsis. The T_reg_/CD4^+^ percentages on day 1 were similar between survivors and non-survivors in this work. This might suggest that the T_reg_ differentiation from naïve CD4^+^ T helper lymphocytes was similar between survivors and non-survivors. However, CD4^+^ lymphocyte counts in survivors were higher than those in non-survivors on day 1. Furthermore, serial T_reg_/CD4^+^ percentages and circulatory T_reg_ lymphocyte counts in survivors and non-survivors did not differ between day 1 and day 7. These results indicate that the benefit of higher circulatory T_reg_ lymphocyte counts in survivors may be due to higher circulatory CD4^+^ lymphocyte counts.

Nascimento et al. [18] reported that treatment of mice with antibody for T_reg_ cells reduced the frequency of T_reg_ cells, restored CD4^+^ T-cell proliferation, reduced the levels of bacteria in spleen, and markedly improved survival in *Legionella pneumophila* infection. Compared with non-survivors with severe sepsis, survivors had higher T_reg_, Th1, Th17 and total CD4^+^ lymphocyte counts in this work. Our results differ from Nascimento et al.’s study. Patients with lower T_reg_ cells did not have higher CD4^+^ T cells in severe sepsis. Perhaps Nascimento et al.’s research investigated the role of T_reg_ cells in the immune response to secondary infection and our study analyzed a primary infection. Plasma IL-6 level in survivors with severe sepsis was lower than that in non-survivors [19, 20]. In this work, total T_reg_ cells in survivors were higher. Addition of human T_reg_ cells to polymorphonuclear neutrophil suspension resulted in a significant decrease of IL-6 production [21]. Thus, lower plasma IL-6 levels in survivors may be due to higher T_reg_ cell counts.

Th17 lymphocytes play an important role in inflammation and host defense [22]. The preoperative expression of IL-17 mRNA from PBMCs was significantly lower in patients who developed sepsis after radical cystectomy [23]. This work first found that the circulatory Th17 lymphocyte counts on day 1 in survivors with severe sepsis were higher than those in non-survivors. Again, the higher circulatory Th17 lymphocyte counts may relate to higher CD4^+^ lymphocyte counts in survivors because Th17/CD4^+^ percentages between survivors and non-survivors were similar. Serial cell analysis also found that not only Th17/CD4^+^ percentages but also circulatory Th17 lymphocyte counts in survivors were significantly increased after 6 days. Based on the above results, we can reasonably hypothesize important roles of total CD4^+^ lymphocyte counts and Th17 lymphocyte differentiation in helping survival in severe sepsis.

Lymphocyte apoptosis is increased in CD4^+^ and CD8^+^ T cells in sepsis as compared to non-sepsis patients [24]. The CD4^+^ cells might facilitate the early clearance of bacteria by regulating neutrophil function, possibly through an IFN-γ-dependent mechanism [25]. Thus, patients with severe sepsis might benefit from increased CD4^+^ and CD8^+^ lymphocytes. However, the CD4^+^ and CD8^+^ T-cell counts in non-survivors with severe sepsis after major surgery were approximately twice as high as those in survivors [26]. Monserrat et al. [27] did not find a difference in CD3^+^CD4^+^ and CD3^+^CD8^+^ cell counts on ICU admission day between survivors and non-survivors with septic shock. These findings are in significant contrast to our results. In this work, the percentages and circulatory cell counts of CD4^+^ and CD8^+^ lymphocytes in survivors were higher than those in non-survivors on day 1. Furthermore, CD4^+^ and CD8^+^ lymphocyte percentages in PBMCs of survivors were significantly increased after 6 days. More CD4^+^ T lymphocytes may help patients with severe sepsis to survive. Our results were also similar to Chen et al.’s study [28]. They found that the percentage of CD4^+^ T lymphocytes in PBMCs was lower in the non-survivors with septic shock, compared with the survivors. There was no difference in the percentage of CD8^+^ T lymphocytes between the non-survivor and survivor groups. Based on the above findings, the low CD4^+^ and CD8^+^ lymphocyte counts may be a consequence of severe sepsis but also suggest a pre-existing condition of low CD4^+^ lymphocyte counts tending to give a poor outcome.

In this work, non-survivors were associated with lower Th1/CD4^+^ and circulatory Th1 lymphocyte counts at the same time. These results were similar to the concept that lack of a shift from Th1 to Th2 response increases survival among patients with sepsis [29]. Unlike those studies measuring Th1 (IFN-γ or TNF-α) and Th2 (IL-4 or IL-10) cytokine levels [19, 20, 30, 31], our study directly measured T-cell phenotypes and first demonstrated that increased Th1 lymphocyte differentiation was associated with greater survival.

There was one limitation in this work. Although the CD25 molecule is expressed on all activated peripheral CD4^+^ T cells, T_reg_ cells were detected with CD4^+^ and high CD25^+^ expression [32], not with CD4^+^ and positive forkhead box P3 (Foxp3). Naturally occurring and induced T_reg_ cells express the signal transduction molecule, Foxp3, and the level of Foxp3 expression in T cells is related to the T_reg_-suppressive activity [33]. However, Foxp3 expression in humans, unlike mice, may not be specific for cells with a regulatory phenotype and may be only a consequence of activation status [34]. Even though CD4^+^CD25^+high^ cells are not specific for T_reg_ cells, CD4^+^CD25^+high^ cells could be regarded as T_reg_ cells in part.

In conclusion, this work showed that T_reg_ lymphocytes were not directly associated with mortality in severe sepsis. High circulatory Th17 and T_reg_ lymphocyte counts in survivors might be due to high numbers of CD4^+^ lymphocytes. Increased Th17 differentiation might be associated with survival in patients with severe sepsis. Under present support systems and treatment guidelines, total CD4^+^ cell counts play a role influencing the mortality of sepsis patients and a trend toward to Th1 differentiation might help patients with severe sepsis to survive.
